# Therapeutic Potential of Regorafenib in Cisplatin-Resistant Bladder Cancer with High Epithelial–Mesenchymal Transition and Stemness Properties

**DOI:** 10.3390/ijms242417610

**Published:** 2023-12-18

**Authors:** Feng-Che Kuan, Jhy-Ming Li, Yun-Ching Huang, Shun-Fu Chang, Chung-Sheng Shi

**Affiliations:** 1 Graduate Institute of Clinical Medical Sciences, College of Medicine, Chang Gung University, Taoyuan 33302, Taiwan; 8902029@cgmh.org.tw; 2 Division of Hematology and Oncology, Department of Medicine, Chang Gung Memorial Hospital Chiayi Branch, Chiayi 61363, Taiwan; 3 Department of Animal Science, National Chiayi University, Chiayi 60004, Taiwan; jml@mail.ncyu.edu.tw; 4 Division of Urology, Department of Surgery, Chang Gung Memorial Hospital Chiayi Branch, Chiayi 61363, Taiwan; dr5326@cgmh.org.tw; 5 Department of Medicine, College of Medicine, Chang Gung University, Taoyuan 33302, Taiwan; 6 Department of Medical Research and Development, Chang Gung Memorial Hospital Chiayi Branch, Chiayi 61363, Taiwan; 7 Center for General Education, Chiayi Chang Gung University of Science and Technology, Chiayi 61363, Taiwan; 8 Division of Colon and Rectal Surgery, Department of Surgery, Chang Gung Memorial Hospital Chiayi Branch, Chiayi 61363, Taiwan

**Keywords:** bladder cancer, cisplatin resistance, epithelial–mesenchymal transition, regorafenib, stemness

## Abstract

Bladder cancer is becoming one of the most common malignancies across the world. Although treatment strategy has been continuously improved, which has led to cisplatin-based chemotherapy becoming the standard medication, cancer recurrence and metastasis still occur in a high proportion of patients because of drug resistance. The high efficacy of regorafenib, a broad-spectrum kinase inhibitor, has been evidenced in treating a variety of advanced cancers. Hence, this study investigated whether regorafenib could also effectively antagonize the survival of cisplatin-resistant bladder cancer and elucidate the underlying mechanism. Two types of cisplatin-resistant bladder cancer cells, T24R1 and T24R2, were isolated from T24 cisplatin-sensitive bladder cancer cells. These cells were characterized, and T24R1- and T24R2-xenografted tumor mice were created to examine the therapeutic efficacy of regorafenib. T24R1 and T24R2 cells exhibited higher expression levels of epithelial–mesenchymal transition (EMT) and stemness markers compared to the T24 cells, and regorafenib could simultaneously inhibit the viability and the expression of EMT/stemness markers of both T24R1 and T24R2 cells. Moreover, regorafenib could efficiently arrest the cell cycle, promote apoptosis, and block the transmigration/migration capabilities of both types of cells. Finally, regorafenib could significantly antagonize the growth of T24R1- and T24R2-xenografted tumors in mice. These results demonstrated the therapeutic efficacy of regorafenib in cisplatin-resistant bladder cancers. This study, thus, provides more insights into the mechanism of action of regorafenib and demonstrates its great potential in the future treatment of cisplatin-resistant advanced bladder cancer patients.

## 1. Introduction

Bladder cancer is one of the most common malignancies across the world, with the total number of newly established cases and deaths being high every year, affecting more males than females [1,2]. Bladder cancer can be classified into muscle-invasive (MIBC) and non-muscle-invasive subtypes (NMIBC). Although NMIBC accounts for a high proportion of clinically diagnosed patients, up to approximately 70%, patients with MIBC have a poor prognosis [3,4]. Currently, the standard clinical treatment strategy for MIBC is radical cystectomy surgery in combination with cisplatin-based neoadjuvant chemotherapy. However, a considerable number of patients still have cancer recurrence and metastasis and might die within 5 years [4,5,6]. This may be primarily because these patients (~50%) are unable to respond adequately to cisplatin cytotoxicity due to side-effects (renal dysfunction), drug resistance, and other unknown reasons [4,7,8]. In fact, in clinical practice, only ~25% of patients with MIBC can receive cisplatin-based neoadjuvant chemotherapy [9]. Therefore, many other strategies have been investigated for improving existing drugs and the development of novel drugs, some of which are in different stages of clinical trials, such as (i) three dosing cycles of methotrexate, vinblastine, doxorubicin, and cisplatin (MVAC); (ii) immune checkpoint inhibitors; and (iii) drug carrier designs (antibody, nanomaterials, or tumor-specific molecules) for targeted therapy [9,10,11,12]. Additionally, it has been suggested that more effort should be further taken to understand the molecular and genetic profiles of MIBC, especially with regard to cisplatin resistance.

Drug resistance development in cancer is the greatest obstacle to curing patients because it greatly increases the possibility of cancer recurrence and metastasis [13]. The development of drug resistance in cancer is proposed to be associated with its epithelial-mesenchymal plasticity (EMP), which is the ability of cancer cells to make decisions regarding promoting/reversing their epithelial–mesenchymal transition (EMT), which subsequently affects their stemness property [13,14]. EMT is a critical step in the initiation of cancer metastasis, as changes in E-cadherin/N-cadherin levels affect cell–cell junction and cytoskeleton remodeling. Previous studies have found that the enhanced expression of stemness genes, e.g., SOX2, c-MYC, KLF4, OCT4, and Nanog, can reprogram somatic cells into induced pluripotent stem cells [15,16]. Moreover, the upregulation of these stemness markers in cancer cells has also been evidenced to initiate cancer stem cell development, which can subsequently increase the resistance of these cancer cells to clinical drugs and metastatic capability [14]. The molecular mechanisms underlying the development of resistance to cisplatin-based neoadjuvant chemotherapy in patients with MIBC remain unclear. Some reports suggest that the EMP status of bladder cancer could affect its metastatic and stemness properties [17,18,19]. Furthermore, cancer stem cell-like phenotypic transformation in bladder cancer may be associated with the EMT and dedifferentiation transitions, as well as the hypoxic microenvironmental conditions [19,20]. Hence, this study proposed the need for an improved understanding of the EMP effect, namely EMT and stemness promotion, their role in the development of cisplatin resistance in bladder cancer cells, and subsequent therapeutic studies.

The central role of protein kinases in controlling various cell functions, such as proliferation, migration, and survival, has been well-documented. Dysfunction of these kinases might directly initiate disease development, including cancer. Accordingly, protein kinases are considered important targets for cancer therapy; a large number of kinase inhibitor drugs have subsequently been extensively developed and approved by the United States Food and Drug Administration [21,22]. Regorafenib is a first-line clinical drug against various cancers because of its broad-spectrum application in blocking the activity of multiple kinases [23,24]. Regorafenib antagonizes the angiogenesis, metastasis, and abnormal growth of cancers by blocking their respective kinase activity, for example, vascular endothelial growth factor receptors (VEGFR), receptor tyrosine kinases, and so on [23,24]. Currently, regorafenib is used mainly for the treatment of advanced/metastatic stomach, colorectal, and liver cancers [23,24,25]. Many clinical trials of regorafenib in patients with advanced angiosarcoma and pancreatic and biliary tract cancers are also ongoing [26,27,28]. The progression of bladder cancer has also been associated with the uncontrolled activities of various kinases [29,30]. Accumulating evidence has also indicated that different kinases, including epidermal growth factor receptor (EGFR), HER2, and/or nuclear factor-κ B, are correlated with the development of cisplatin resistance in bladder cancers [31]. Recently, the cancer-killing efficacy of regorafenib has been evidenced in bladder cancer, which has no obvious drug resistance [29,30]. However, whether regorafenib can also be used to specifically kill cisplatin-resistant bladder cancer is unknown.

In this study, the therapeutic potential of regorafenib in cisplatin-resistant bladder cancer was examined, and the underlying mechanism was elucidated. We isolated two cisplatin-resistant bladder cancer cells, i.e., T24R1 and T24R2, from T24 cisplatin-sensitive bladder cancer cells and found noticeably higher levels of EMT and stemness markers in these two cells than the T24 cells. Moreover, regorafenib significantly inhibited the survival, transmigration/migration, and tumor pathology of T24R1 and T24R2 cells, as well as T24R1- and T24R2-xenografted tumors; this was also accompanied by a decrease in EMT and stemness markers expressions in T24R1 and T24R2 cells.

## 2. Results

**Figure 1:**
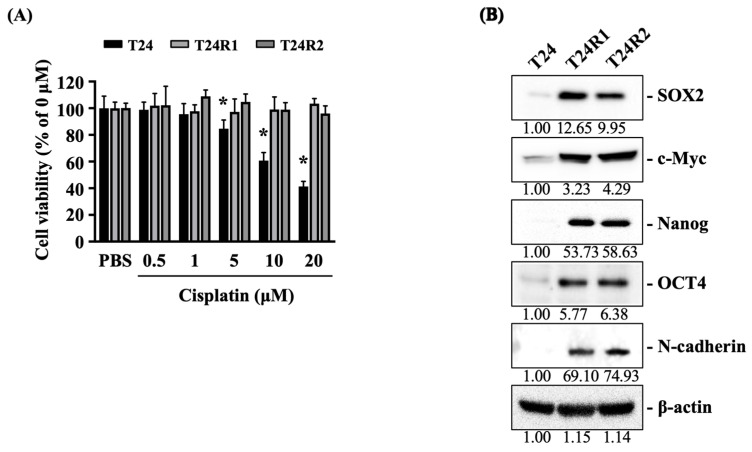
Cisplatin-resistant bladder cancer cells, T24R1 and T24R2, exhibited higher expression of stemness and EMT markers. (**A**) T24, T24R1, and T24R2 cells were treated with PBS to serve as controls or treated with 0.5, 1, 5, 10, and 20 μM of cisplatin for 24 h and their viabilities were examined by CCK-8 assay. (**B**) T24, T24R1, and T24R2 cells were cultured for 24 h and then their expressions of stemness and EMT markers were examined by Western blot. The experiments were repeated independently at least three times. * *p* < 0.05 vs. untreated control (0 h).

Cisplatin-resistant bladder cancer cells, T24R1 and T24R2, exhibited higher expression of stemness and EMT markers. Cisplatin-resistant bladder cancer cells, T24R1 and T24R2, were selected from cisplatin-sensitive bladder cancer T24 cells exposed to serial concentrations of cisplatin (0.5~50 μM). After that, the cisplatin cytotoxicity to T24, T24R1, and T24R2 cells was examined again (CCK-8 assay) by treating the cells with 0.5, 1, 5, 10, and 20 μM of cisplatin for 24 h. Cisplatin significantly decreased the viability of T24 cells in a dose-dependent manner (from 5 to 20 μM) (Figure 1A), but did not affect the survival of T24R1 and T24R2 cells, even at higher doses (Figure 1A). Moreover, the examination of stemness (SOX2, c-Myc, Nanog, and OCT-4) and EMT (N-cadherin) markers of these three types of cells revealed a significantly higher expression of stemness/EMT markers in T24R1 and T24R2 cells than T24 cells (Figure 1B, Figures S1 and S4).

**Figure 2:**
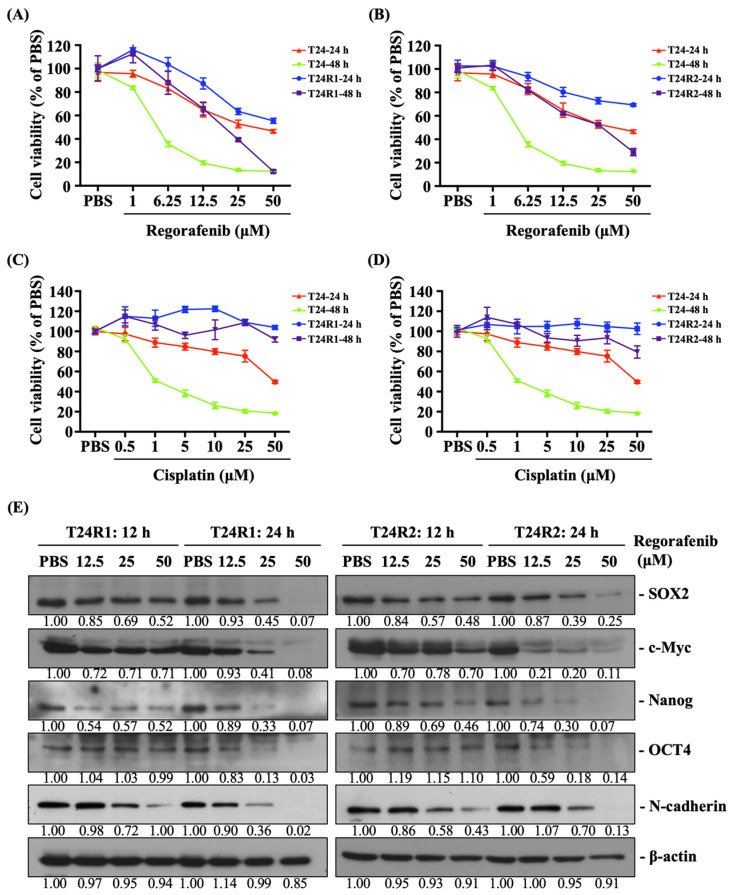
Regorafenib decreased the viability of T24R1 and T24R2 cells and inhibited their expression of stemness and EMT markers. (**A**,**B**,**E**) T24, T24R1, and T24R2 cells were treated with PBS to serve as controls or (**A**,**B**,**E**) treated with regorafenib at 1, 6.25, 12.5, 25, and 50 μM or (**C**,**D**) treated with cisplatin at 1, 5, 10, 25, and 50 μM for 12, 24, and 48 h, and their (**A**–**D**) viabilities and (**E**) expressions of stemness and EMT markers were examined by CCK-8 assay and Western blot, respectively. The experiments were repeated independently at least three times.

Regorafenib decreased the viability of T24R1 and T24R2 cells and inhibited their expressions of stemness/EMT markers. Regorafenib has been used for the treatment of patients with various advanced cancers [23,24,25]. Hence, we examined if it could also kill cisplatin-resistant bladder cancer cells, i.e., T24R1 and T24R2, and stall the expression of stemness and EMT markers. Both cells were treated with PBS to serve as controls or treated with regorafenib at 1, 6.25, 12.5, 25, and 50 μM for 12, 24, and 48 h, and their viability (24 h and 48 h) and the expression of stemness (SOX2, c-Myc, Nanog, and OCT-4) and EMT (N-cadherin) markers (12 h and 24 h) were examined by CCK-8 assay and Western blot, respectively. Regorafenib could significantly decrease the viability of both T24R1 and T24R2 cells (Figure 2A,B) and inhibit their expression of stemness and EMT markers (Figure 2E, Figures S2 and S5) in a dose- (from 6.25 to 50 μM) and time-dependent manner compared to the PBS-treated cells. For comparison, T24 cells were also treated with regorafenib at the same doses and three types of cells were treated with cisplatin at 1, 5, 10, 25, and 50 μM for 24 h and 48 h and then their viability was examined by CCK-8 assay. It was shown that the cytotoxicity of regorafenib in T24 cells is stronger than that of the T24R1 and T24R2 cells at the same doses and durations (Figure 2A,B). Further, as expected, cisplatin did not affect the viability of both T24R1 and T24R2 cells, but significantly inhibited the survival of T24 cells in a dose- and time-dependent manner (Figure 2C,D).

**Figure 3:**
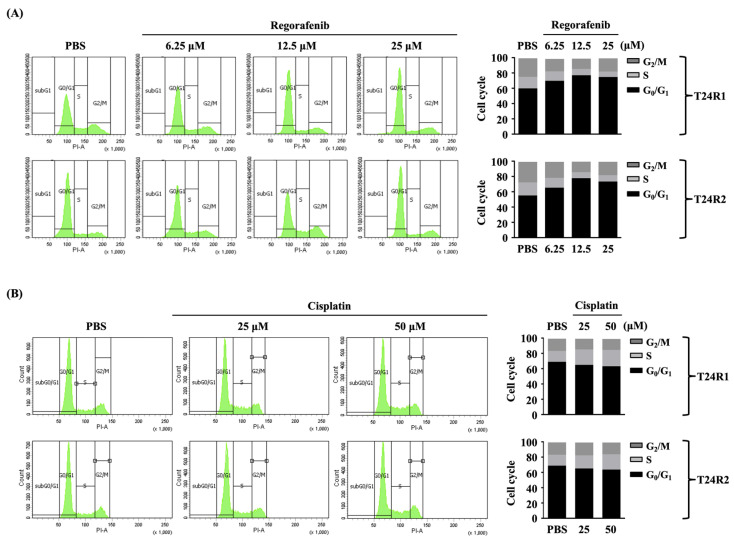
Regorafenib induced cell cycle arrest at the G0/G1 phase in both T24R1 and T24R2 cells. (**A**,**B**) Both cells were treated with PBS to serve as controls or treated with (**A**) regorafenib at 6.25, 12.5, and 25 μM or (**B**) cisplatin at 25 and 50 μM for 12 h and then their cell cycle distributions were examined by PI stain. The experiments were repeated independently at least three times.

**Figure 4:**
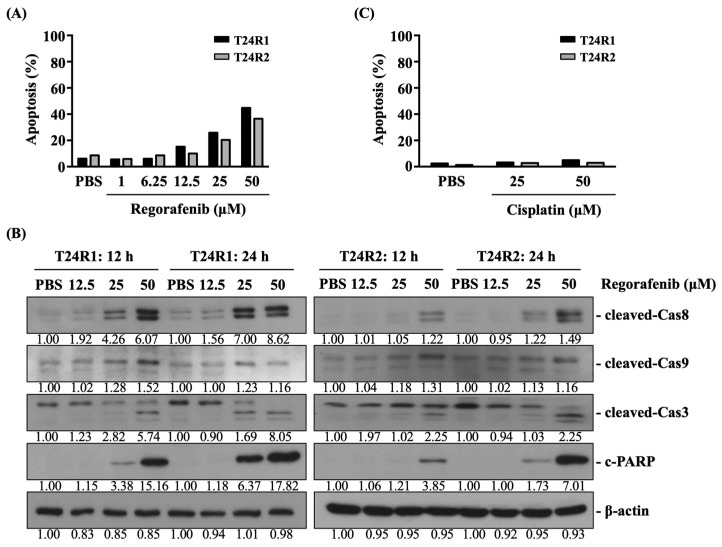
Regorafenib initiated the apoptosis in both T24R1 and T24R2 cells. (**A**–**C**) Both cells were treated with PBS to serve as controls or treated with (**A**,**B**) regorafenib at 1, 6.25, 12.5, 25, and 50 μM or with (**C**) cisplatin at 25 and 50 μM for 12 and 24 h, and their apoptosis (**A**,**C**) and expressions of cleaved caspase 8, 9, and 3 and PARP (**B**) were examined by PI/Annexin V stain and Western blot, respectively. The experiments were repeated independently at least three times.

Regorafenib induced cell cycle arrest at the G0/G1 phase and initiated the apoptosis of T24R1 and T24R2 cells. Next, we determined if regorafenib causes T24R1 and T24R2 cell death by controlling cell cycle distribution and apoptosis. Both cells were treated with PBS to serve as controls or treated with regorafenib at 1, 6.25, 12.5, 25, and 50 μM for 12 and 24 h and their cell cycle distribution (12 h); apoptosis (12 h); and cleaved caspase 8, 9, and 3 and poly (ADP-ribose) polymerases (PARP) levels (12 h and 24 h) were examined by PI stain, PI/Annexin V stain, and Western blot, respectively. For comparison, both types of cells were also treated with cisplatin at 25 and 50 μM for 12 h. Regorafenib dose-dependently causes an increase in the number of cells (T24R1 and T24R2) in the G0/G1 phase and a decrease in their number in S and G2/M phases of the cell cycle (from 6.25 to 25 μM) (Figure 3A). Moreover, regorafenib also initiated apoptosis in T24R1 (from 12.5 to 50 μM) and T24R2 (from 25 to 50 μM) cells in a dose-dependent manner (Figure 4A), which was accompanied with increasing levels of cleaved caspase 8, 9, and 3 and PARP, compared to the PBS-treated cells (Figure 4B, Figures S3 and S6). However, cisplatin did not affect the cell cycle distribution (Figure 3B) and apoptosis (Figure 4C) of both T24R1 and T24R2 cells.

**Figure 5:**
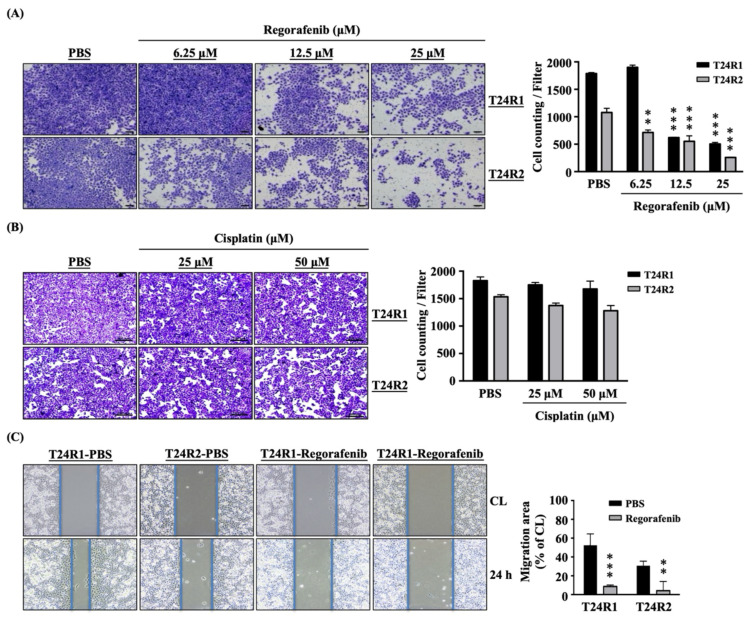
Regorafenib inhibited the transmigration and migration capabilities of both T24R1 and T24R2 cells. (**A**) Both cells were treated with PBS to serve as controls or treated with (**A**) regorafenib at 6.25, 12.5, and 25 μM or (**B**) cisplatin at 25 and 50 μM for 24 h and then their transmigration capabilities were examined by a transwell assay. (**C**) Both cells were untreated (CL, 0 h) or treated with PBS or regorafenib at 12.5 μM for 24 h and then their migration capabilities were examined by a wound-healing assay. The experiments were repeated independently at least three times. ** *p* < 0.01 vs. PBS-treated cells. *** *p* < 0.001 vs. PBS-treated cells. Scale bars in (**A**,**B**) are 100 μm and 200 μm, respectively.

Regorafenib inhibited the transmigration and migration capabilities of both T24R1 and T24R2 cells. T24R1 and T24R2 cells were treated with PBS to serve as controls or treated with regorafenib at 6.25, 12.5, and 25 μM for 24 h and then their transmigration capability was examined by a transwell assay. For comparison, both types of cells were also treated with 25 and 50 μM of cisplatin for 24 h. Regorafenib at higher concentrations (12.5 and 25 μM) decreased the transmigration capability of T24R1 and T24R2 cells compared to the PBS-treated cells (Figure 5A). However, at 6.25 μM, regorafenib could only decrease the transmigration capability of T24R2 cells, but not T24R1 cells. Cisplatin did not affect the transmigration capability (Figure 5B) of both T24R1 and T24R2 cells. Both cells were untreated (CL, 0 h) or treated with PBS or regorafenib at 12.5 μM for 24 h and then their migration capability was examined by a wound-healing assay. It was shown that regorafenib could significantly block the migration capability of both cells compared to the PBS-treated cells (Figure 5C).

**Figure 6:**
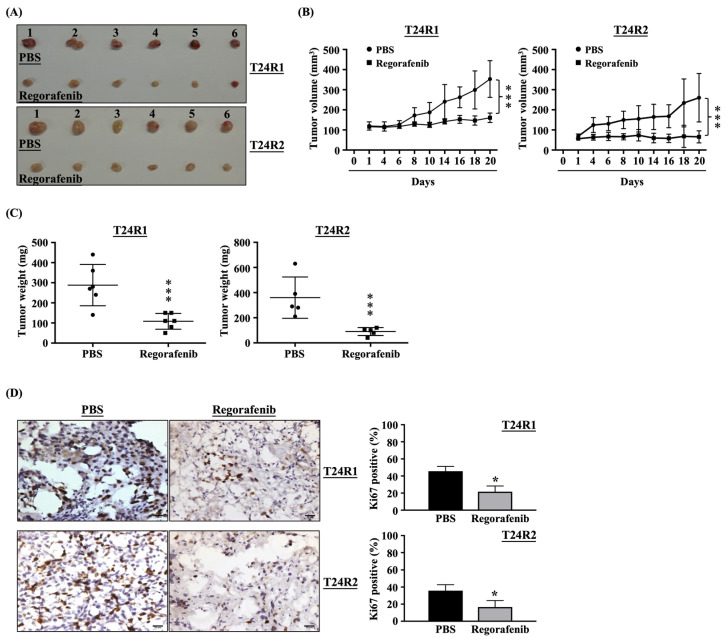

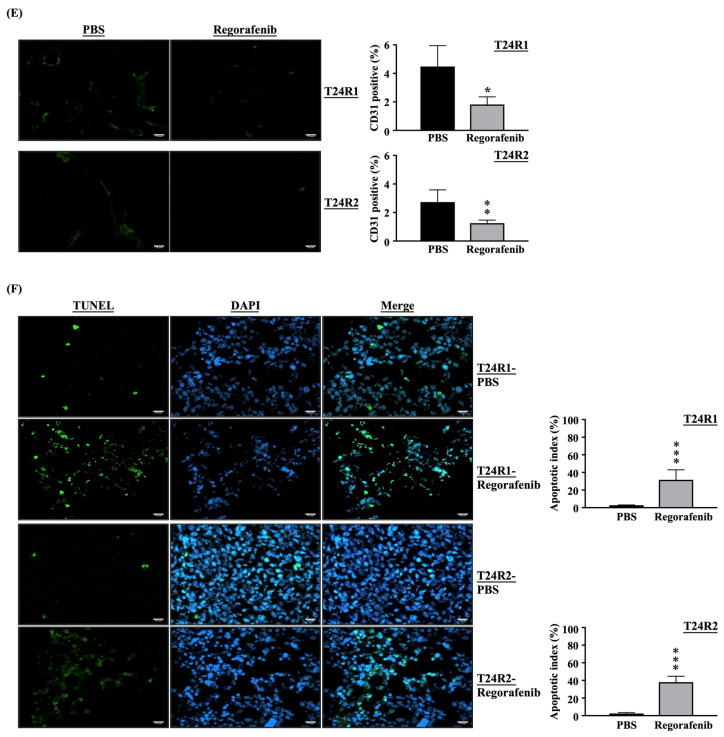
Regorafenib inhibited tumor growth and Ki67/CD31 expression and initiated apoptosis in the T24R1- and T24R2-xenografted mice. T24R1 and T24R2 cells were subcutaneously injected into nude mice to initiate the growth of xenograft tumors. After tumor growth to ~100 mm^3^, T24R1- and T24R2-xenografted mice were intraperitoneal injected with PBS or regorafenib (20 mg/kg) for 20 consecutive days. (**B**,**C**) The volume (**B**) and weight (**C**) of T24R1- and T24R2-xenografted tumors were measured finally and the tumor pathology, such as the levels of (**D**) proliferation (Ki-67 stain), (**E**) angiogenesis (CD31 stain), and (**F**) apoptosis (TUNEL stain), were examined by the immunohistochemical stain of excised tumors. * *p* < 0.05, ** *p* < 0.01 vs. (**D**,**E**) PBS-treated group. *** *p* < 0.001 vs. (**B**,**C**,**F**) PBS-treated group. Scale bars in (**D**–**F**) are 20 μm.

Regorafenib inhibited tumor growth and Ki67/CD31 expression and initiated apoptosis in the T24R1- and T24R2-xenografted mice. To examine the exact antitumor efficacy of regorafenib in vivo, T24R1 and T24R2 cells were subcutaneously injected into nude mice to initiate xenograft tumor growth. After tumor growth to ~100 mm^3^, T24R1- and T24R2-xenografted mice were intraperitoneally injected with PBS or regorafenib (20 mg/kg) for 20 consecutive days. It was shown that regorafenib significantly blocks the growth of T24R1- and T24R2-xenografted tumors compared to PBS-treated mice (Figure 6A and Figure S7). Moreover, the volume (Figure 6B) and weight (Figure 6C) of T24R1- and T24R2-xenografted tumors were also reduced by approximately 60% and 70%, respectively. To assess tumor pathology, the levels of cell proliferation (Ki-67 stain), angiogenesis (CD31 stain), and apoptosis (TUNEL stain) were examined by the immunohistochemical stain of excised tumors. Regorafenib treatment significantly reduced the level of Ki-67 (Figure 6D) and CD31 (Figure 6E) in T24R1- and T24R2-xenografted tumors, which indicated that regorafenib could block proliferation and angiogenesis in tumors. Moreover, more TUNEL-positive levels were found in the regorafenib-treated groups, indicating that regorafenib could further promote apoptosis in T24R1- and T24R2-xenografted tumors (Figure 6F).

## 3. Discussion

This study demonstrated that regorafenib could also effectively antagonize the survival of cisplatin-resistant bladder cancer cells. Our systemic experiments revealed that (i) the isolated cisplatin-resistant bladder cancer cells, T24R1 and T24R2, are indeed highly resistant to cisplatin cytotoxicity (even at 20 μM); (ii) T24R1 and T24R2 cells exhibited a higher expression of stemness and EMT markers compared to those in T24 cells; (iii) regorafenib arrests the cell cycle in the G0/G1 phase and initiates apoptosis to kill T24R1 and T24R2 cells, accompanied by a decreased expression of stemness and EMT markers; (iv) regorafenib also blocked the transmigration and migration capability of T24R1 and T24R2 cells; (v) regorafenib antagonized T24R1- and T24R2-xenografted tumor growth, with a concomitant inhibition of proliferation and angiogenesis and promotion of apoptosis.

Regorafenib is a multi-kinase inhibitor that significantly blocks the kinase activity associated with angiogenesis, abnormal proliferation, apoptosis resistance, and metastasis within the tumor microenvironment [23,24]. Regorafenib is remarkably efficient; therefore, it has often been used in treating patients with advanced cancers [23,24,25]. In addition to being a first-line medication, regorafenib is also considered as the dominant candidate in salvage-line medication [32,33]. The standard systemic medication for metastatic colorectal cancer and metastatic gastrointestinal cancer includes cytotoxic drugs (for example, oxaliplatin or bevacizumab) and/or targeted agents (anti-VEGFR, anti-EGFR antibodies). However, after the failure of these drugs, regorafenib was approved for its efficacy as a salvage agent in treating those patients [32,33,34]. Also, in this condition, regorafenib could be used as monotherapy or combined therapy with other drugs, e.g., trifluridine/tipiracil [34,35,36]. Regorafenib was also recently found to antagonize bladder cancer (TSGH8301 cells) by inhibiting its survival, transmigration/migration capacity, and subsequent tumor growth through different mechanisms [29,30]. Accordingly, in this study, we could further select and isolate two cisplatin-resistant bladder cancer cells from cisplatin-sensitive bladder cancer cells. We demonstrated that regorafenib could greatly antagonize the efficacy of these more aggressive and malignant cells and subsequent tumor growth. Combining the above results from other studies and ours, we propose that regorafenib might be a potentially great therapeutic candidate for first-, second-, and/or even salvage-line medications in treating patients with cisplatin-sensitive/resistant bladder cancers.

The causes of drug resistance development in cancer cells may be mutations or the abnormal expression of genes/proteins, cell death inhibition, a dysfunctional drug metabolism and DNA repair system, and the heterogeneity of the microenvironment. Cisplatin-based neoadjuvant chemotherapy has been considered beneficial and recommended in combination with radical cystectomy as the predominant treatment strategy for patients with MIBC by the National Comprehensive Cancer Network and the European Association of Urology [9,37]. However, although this combined therapy improved all clinical data significantly, more efforts and research into new treatment strategies are unmet needs, as the prognosis of patients remains poor. The resistance of MIBC to the cytotoxicity of cisplatin-based systemic drug therapy is considered the most common cause of this outcome [9,37]. The cytotoxicity of cisplatin results in DNA damage and, hence, blocks proliferation and initiates cell death [8,9]. It has been further suggested that the possible mechanisms of cisplatin resistance development potentially include the abnormal activation of (i) the DNA repair system (such as actin-like 6A and nucleotide excision repair proteins) [38,39]; (ii) downstream signaling molecules (such as EGFR) [7,31]; and (iii) cancer stem cell-like characters (many stemness markers identified in earlier studies and our present study) [17,19,20].

Understanding the cause of drug resistance is important for clinicians to decide on the therapeutic strategy [40]. Our results reveal that the expression levels of stemness (SOX2, c-MYC, Nanog, and OCT4) and EMT (N-cadherin) markers are positively correlated with the cisplatin resistance development of bladder cancer cells. It has been indicated that cancer cells’ epithelial plasticity could decide the direction of epithelial–mesenchymal transition and could, hence, affect the development of stemness properties. Moreover, the EMT and stemness promotion of cancer cells would subsequently increase their drug resistance development and finally lead to their recurrence and metastasis after the patients are cured for a few years [13,14,16,41,42,43]. In this case, the upregulation of EMT- and stemness-related proteins are critical for the increased escape behavior of cancer cells, including the increase in transmigration and migration capabilities of cancer stem cell characteristics [13,14,16,41,42,43]. However, while our data further evidenced that regorafenib could also reduce the expression levels of stemness and EMT markers in T24R1 and T24R2 cells, our results showed a more pronounced cytotoxicity effect of regorafenib on cisplatin-sensitive bladder cancer cells (T24) compared to cisplatin-resistance cells (T24R1 and T24R2). We reasonably proposed that additional resistance mechanisms (as previously described) might exist in both cisplatin-resistant cells. Taken together, in addition to the inhibition of kinase activity, our findings suggested that the inhibition of EMT and stemness could also be a therapeutic mechanism of regorafenib in the treatment of recurrent and metastatic cancers. In addition, the development of cisplatin resistance in bladder cancers might be more complex than expected, thus requiring consideration of additional resistance triggers to develop new drugs and/or improve existing treatment strategies.

The limitations of this study were that (i) no results were available for advanced clinical bladder cancer tissues to support our findings on the upregulation of EMT and stemness promoting resistance; and that (ii) we only obtained the proof of the correlation between expression levels of EMT/stemness markers and drug resistance/therapeutic efficacy of regorafenib, but did not further examine their direct association. Moreover, regorafenib is already a clinical drug for treating various cancer types; therefore, after future clarifications of our limitations, a clinical trial of regorafenib in advanced bladder cancer, especially cisplatin-resistant bladder cancer, is needed.

Regorafenib is a well-researched and widely used clinical drug in treating various advanced cancers, and more clinical trials for other cancer types are underway. Previous studies have identified the cancer-killing capability of regorafenib against bladder cancer cells. Furthermore, a clinical trial (NCT02459119) of regorafenib in patients with bladder cancer appears to be ongoing currently. This study further demonstrates the therapeutic potential of regorafenib in cisplatin-resistant bladder cancer, a commonly occurring type of advanced bladder cancer. Thus, the study findings provide additional insights into the therapeutic mechanism of regorafenib. These provide useful information to clinicians when deciding on medication strategies for (advanced) bladder cancer patients in the future.

## 4. Materials and Methods

### 4.1. Materials

Cisplatin was purchased from Merck (Darmstadt, Germany; Cat. #15663-27-1). Cisplatin was dissolved in normal saline (0.9% NaCl *w*/*w*) at a final concentration of 0.5 mg/mL (1.67 mM), aliquoted, and stored in the dark at 2–8 °C. Regorafenib was purchased from the MedChemExpress (Monmouth Junction, NJ, USA; Cat. #HY-10331). Regorafenib was dissolved in DMSO (Merck, Darmstadt, Germany; Cat. #67-68-5) at a final concentration of 50 mM, aliquoted, and stored in the dark at −80 °C, then added to culture medium, resulting in a final DMSO concentration of 0.1%. The propidium iodide (PI) reagent (Cat. #550825) and PI/Annexin V-Apoptosis Detection Kit (Cat. #556547) were purchased from BD Biosciences (Bedford, MA, USA). The specific antibodies against SOX2 (cat. #23064), c-MYC (Cat. #18583), Nanog (Cat. #3580), OCT4 (Cat. #2750), N-cadherin (Cat. #13116), cleaved caspase 8 (Cat. #9463), 9 (Cat. #7237), 3 (Cat. #9664), c-PARP (Cat. #5625), and β-actin (Cat. #4970) were purchased from Cell Signaling Technology (Danvers, MA, USA). Other materials that were not described were purchased from Sigma (St. Louis, MO, USA).

### 4.2. Cell Culture

Human T24 bladder cancer cells (cisplatin-sensitive) were purchased from the cell bank in the American Type Culture Collection (Rockville, MD, USA; Cat. #HTB-4). T24R1 and T24R2 (cisplatin-resistant) cells were repeatedly exposed to increasing concentrations of cisplatin until the IC_50_ reached 50 µM for 6 mouths. Subsequently, these cisplatin-resistant cells were maintained in a complete medium containing 20 µM of cisplatin to maintain the resistance [44]. All types of cells were cultured in RPMI 1640 medium (Thermo, Waltham, MA, USA; Cat. #11875093) with 10% serum (Thermo, Waltham, MA, USA; Cat. #10270106) and 1% antibiotics (LONZA, Basel, Swiss; Cat #VZA-2012) and incubated in a 37 °C/5% CO_2_ incubator.

### 4.3. Cell Viability

Cell viability was analyzed by the CCK-8 Cell Counting Kit (Roche Applied Science, Penzberg, Germany; Cat. #96992) [44]. Cells were seeded in a 96-well plate at a density of 5×10^3^ cell per well and cultured for 24 h. The cells were then treated with regorafenib in a range from 1 to 50 μM and incubated for 24 h. After stimulation, the cells were treated with a CCK-8 agent and then further incubated for the indicated times. Finally, the absorbance of the cells was measured at 450 nm.

### 4.4. Apoptosis

Cell apoptosis was analyzed by a PI/Annexin V-Apoptosis Detection Kit and flow cytometry [45]. Cells were seeded in a 6-well plate at a density of 4 × 10^5^ cells per well and cultured for 24 h. After that, various concentrations of regorafenib were added to the wells and incubated for 24 h. The cells were detached from the 6-well plate using Trypsin/EDTA and then were double-stained with PI and Annexin V agents provided by an apoptosis detection kit. Finally, the stained cells were analyzed by a flow cytometer and CellQuest Software (BD Biosciences, Bedford, MA, USA).

### 4.5. Western Blot

Protein expression levels were analyzed by Western blot [46]. After treatment, total proteins were extracted and collected from the cells lysed by adding the lysis buffer (Millipore, Darmstadt, Germany) and the protease/phosphatase inhibitor mixture (Roche Applied Science, Penzberg, Germany; Cat. #04906837001). The total proteins were separated (molecular weight) in the SDS-PAGE with upper stacking (4%) and down running (10%) gels and then were transferred onto the nitrocellulose membrane (0.45 µm). The analyzed proteins were treated with specific antibodies and their expression levels were analyzed by an ABI Western-Light chemiluminescent instrument (Applied Biosystems, Foster City, CA, USA). The signal intensities of protein bands from Western blot films were quantified using ImageJ 1.53v software.

### 4.6. Cell Cycle Distribution Analysis

The cell cycle was examined by PI stain (BD Biosciences; Cat. #550825) and flow cytometry [47]. After treatment, the cells were collected and fixed by cold 70% ethanol. Then, the cell membrane permeability was opened by a 0.1% Triton X-100. After that, the cells’ DNA was stained by PI reagent and ribonuclease A simultaneously, and the cell cycle distribution of the stained cells was finally analyzed in a flow cytometer.

### 4.7. Transmigration Assay (Transwell)

The transmigration capability was examined by a transwell chamber (BD Biosciences, Bedford, MA, USA; Cat #353097) [48]. Cells were cultured in the upper inserts and their chemotactic transmigration was promoted to pass through the membrane after stimulation. Finally, the transmigrated cells on the opposite site of the insert were fixed (methanol) and stained (1% Crystal violet) and then the stained cells were counted in a microscope.

### 4.8. Migration Assay (Wound Healing)

The migration capability was examined by a wound-healing assay [49]. Cells were cultured in the two-well culture-insert (Ibidi, Grafelfing, Bavaria, Germany; Cat. #81176). After culturing for 24 h, the inserts were removed and the cells were kept as the control or treated with drugs to compare their cell migration capability. After the indicated times, the migration levels of the cells were photographed and quantified.

### 4.9. Animal Model

All the protocols for the animal study were established and approved by the Animal Care and Use Committee in Chiayi Chang Gung Memory Hospital (approval number: 2018061901; Chiayi, Taiwan) [50]. Surgery (tumor excision) was performed using sodium pentobarbital anesthesia. The immunodeficient mice (CB17-Prkdcscid/NcrCrlBltw (NOD-SCID), male, 8-week-old) were purchased from BioLASCO (Taipei, Taiwan). A volume of 50 μL of cell suspensions (1 × 10^6^ cells in PBS) were mixed with 50 μL of Matrigel solution (Corning, NY, USA; Cat. #CLS356231), and then the mixed solutions (100 μL) were subcutaneously injected into the back of mice to create T24R1- and T24R2-xenograft mice (each group, *n* = 8). After tumor growth to ~100 mm^3^, the mice were intraperitoneally injected with PBS or regorafenib (20 mg/kg) for 20 consecutive days. The tumors were not allowed to grow over 2000 mm^3^. After that, the tumors’ volume/weight was evaluated and the levels of proliferation (Ki-67 stain), angiogenesis (CD31 stain), and apoptosis (TUNEL stain) were examined by immunohistochemical stain.

### 4.10. Statistical Analysis

All the experiments were repeated independently at least 3 times. All the data were analyzed by GraphPad Prism 5 and presented as mean ± standard (SD). Comparisons with two groups were conducted by a two-tailed Student’s *t*-test. A *p* value < 0.05 was recognized as significant.

## Data Availability

The data that support the findings of current study are available from the corresponding author upon reasonable request. The raw data of the Western blot and the tumor size with a ruler are presented as Supplementary Figures S1–S4.

## Supplementary Materials

The following supporting information can be downloaded at: https://www.mdpi.com/article/10.3390/ijms242417610/s1. The supplementary information includes the raw data and quantification plots of the Western blot and the tumor size with a ruler (Supplementary Figures S1–S7).
