# Supplementary material for: Therapeutic Potential of Regorafenib in Cisplatin-Resistant Bladder Cancer with High Epithelial–Mesenchymal Transition and Stemness Properties
Source: Int J Mol Sci. 2023 Dec 18;24(24):17610. doi: 10.3390/ijms242417610 (PMC10743903; doi:10.3390/ijms242417610)
Supplement: Supplementary file 1 [file ijms-24-17610-s001.zip › ijms-2699961-supplementary.pdf]

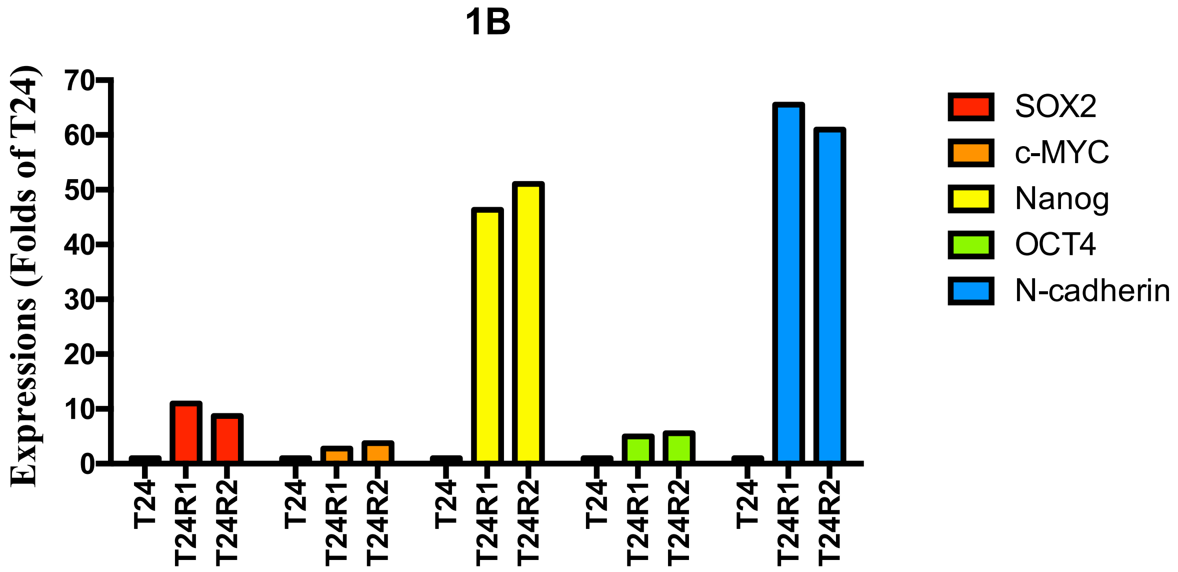

Supplemental Figure 1.
Quantification plot of Figure 1B.

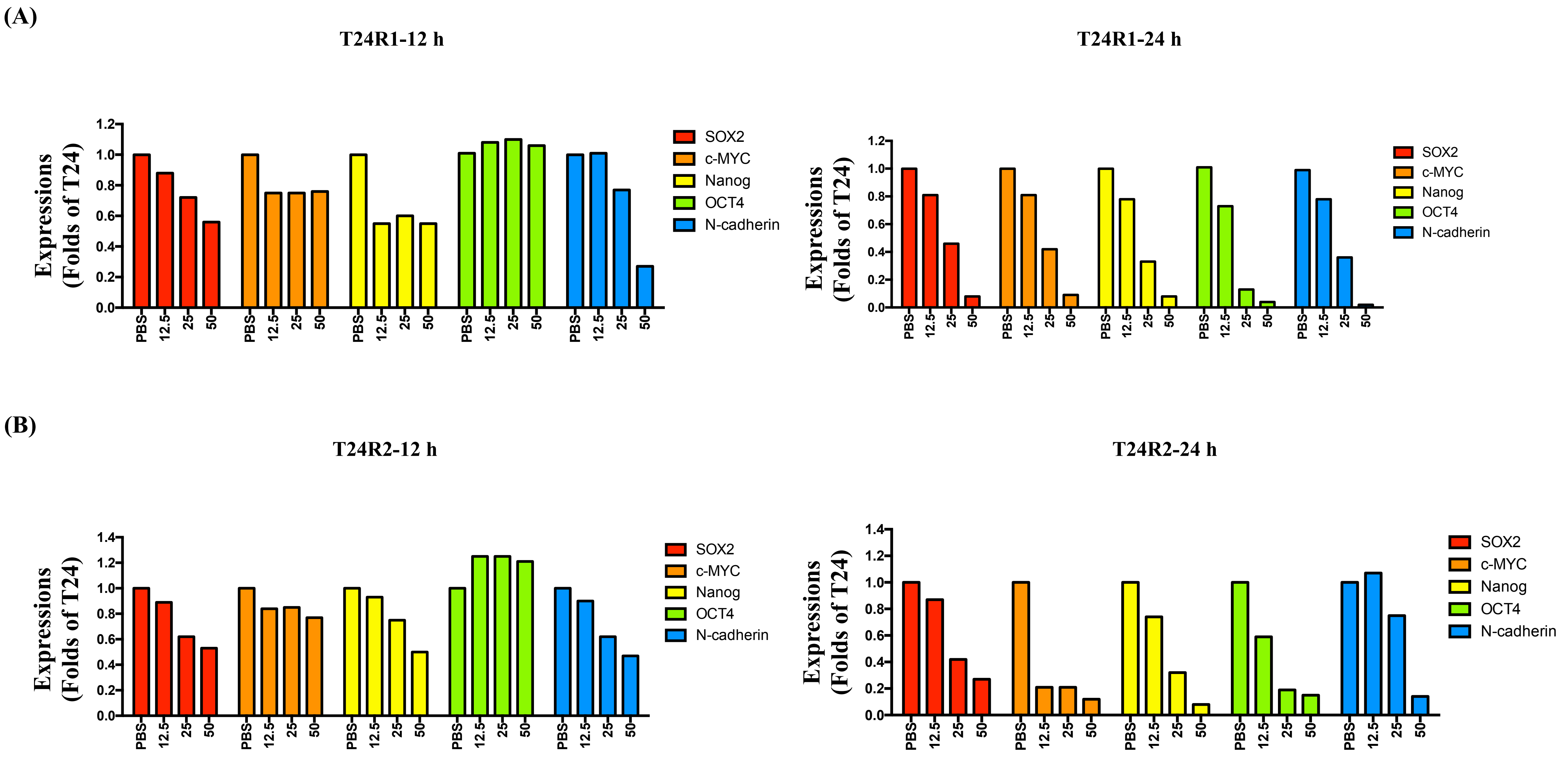

Supplemental Figure 2.
Quantification plot of Figure 2E.

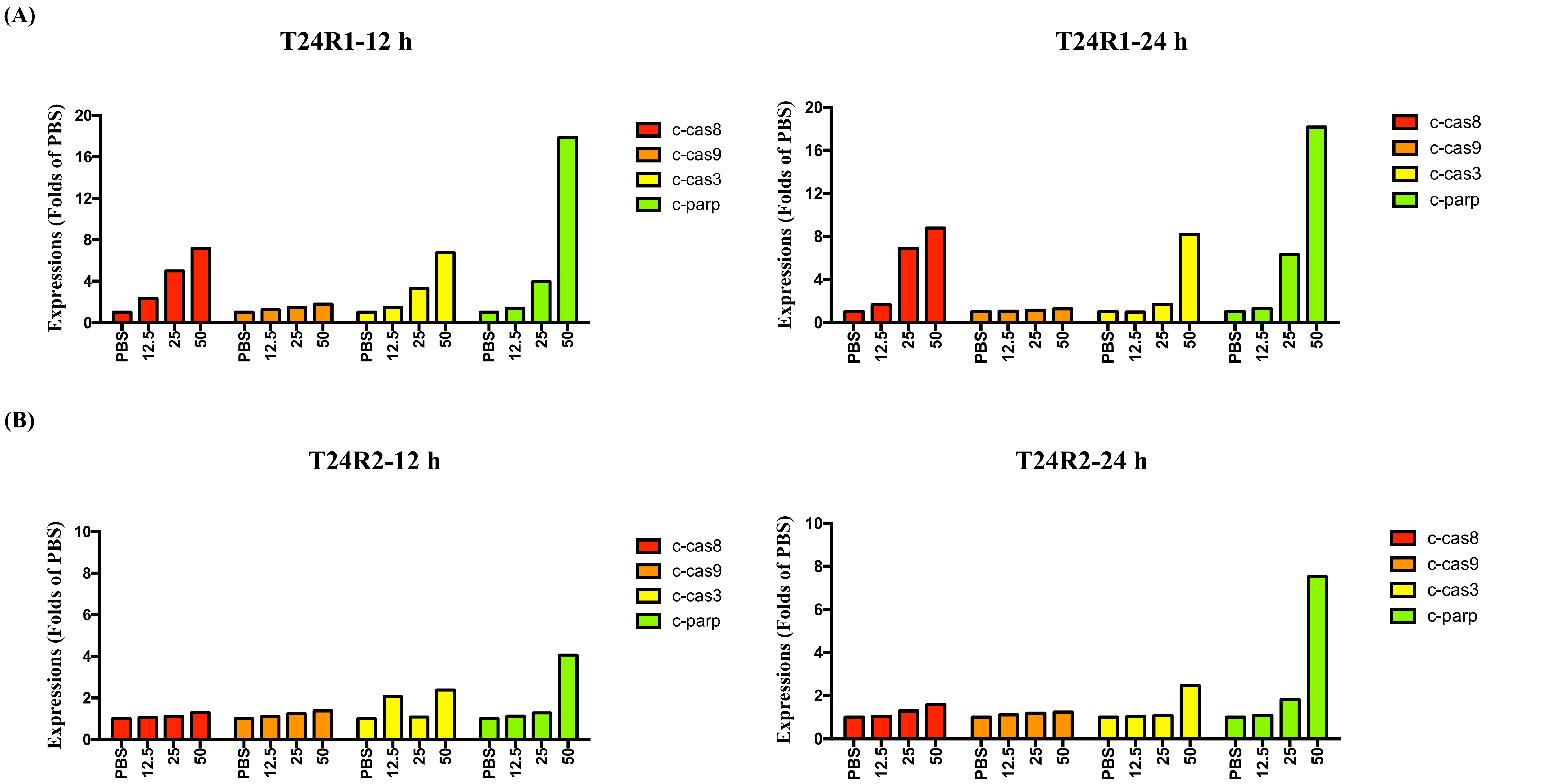

Supplemental Figure 3.
Quantification plot of Figure 4B.

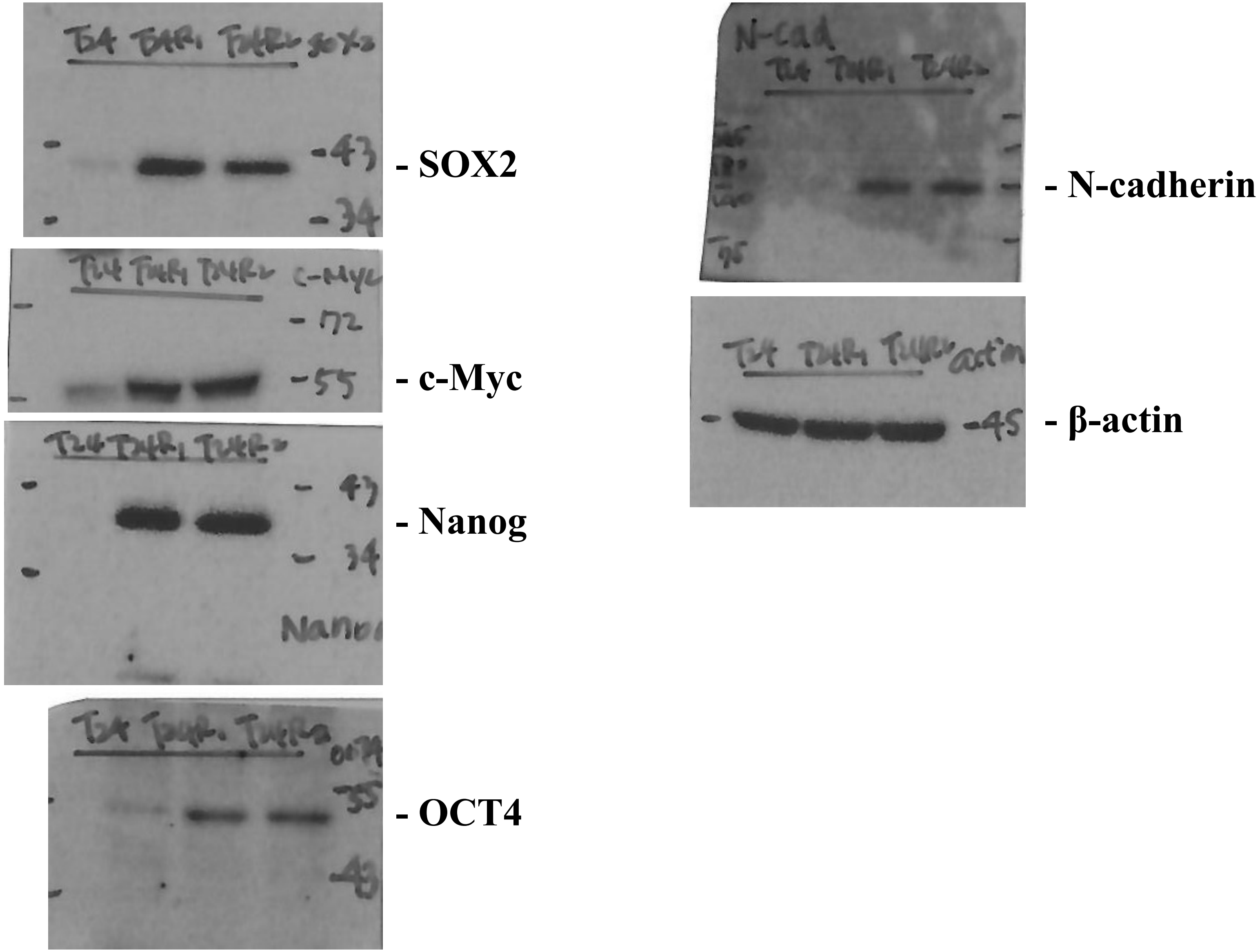

Supplemental Figure 4.
Raw data of Figure 1B.

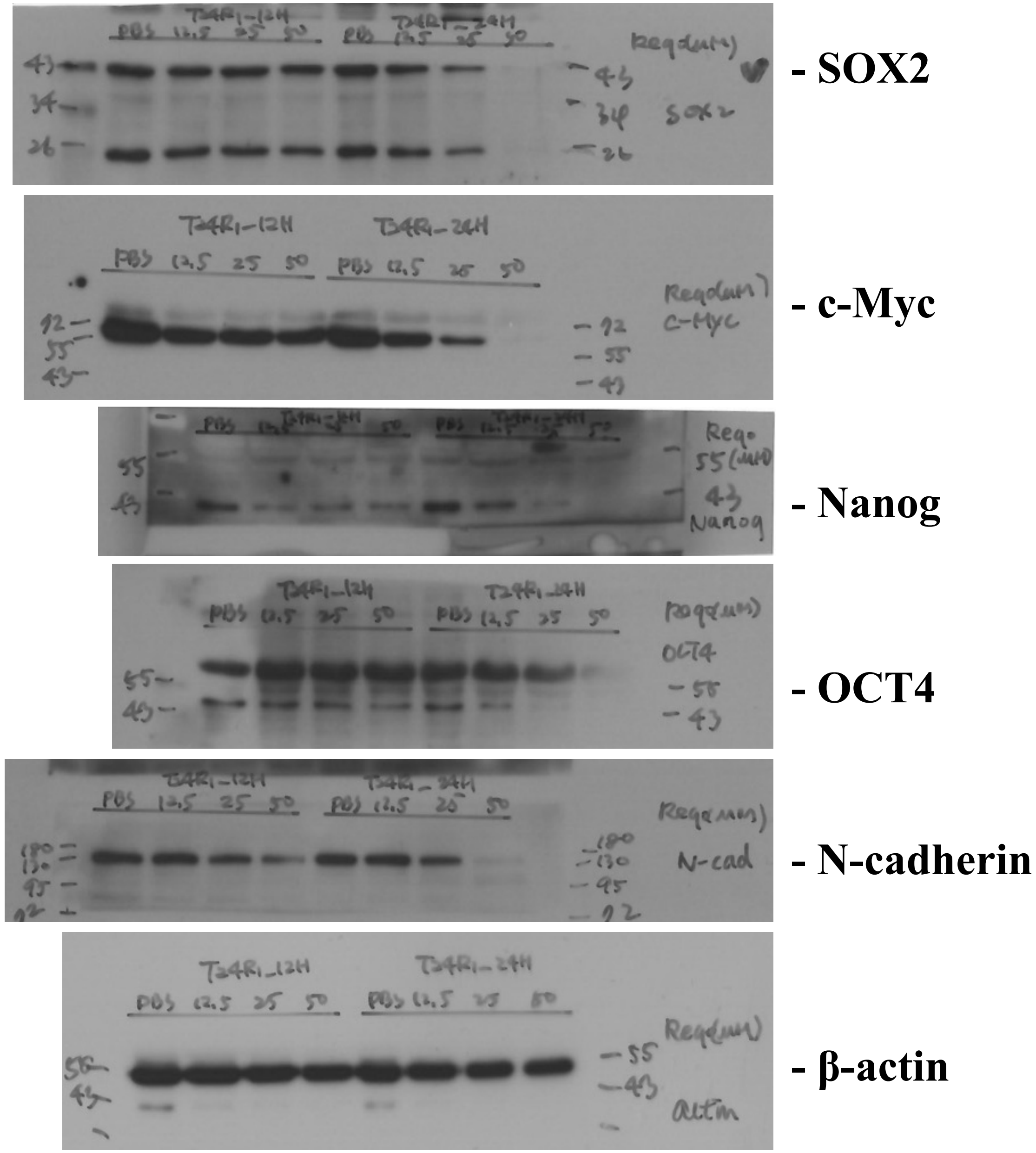

Supplemental Figure 5.
T24R1 raw data of Figure 2E.

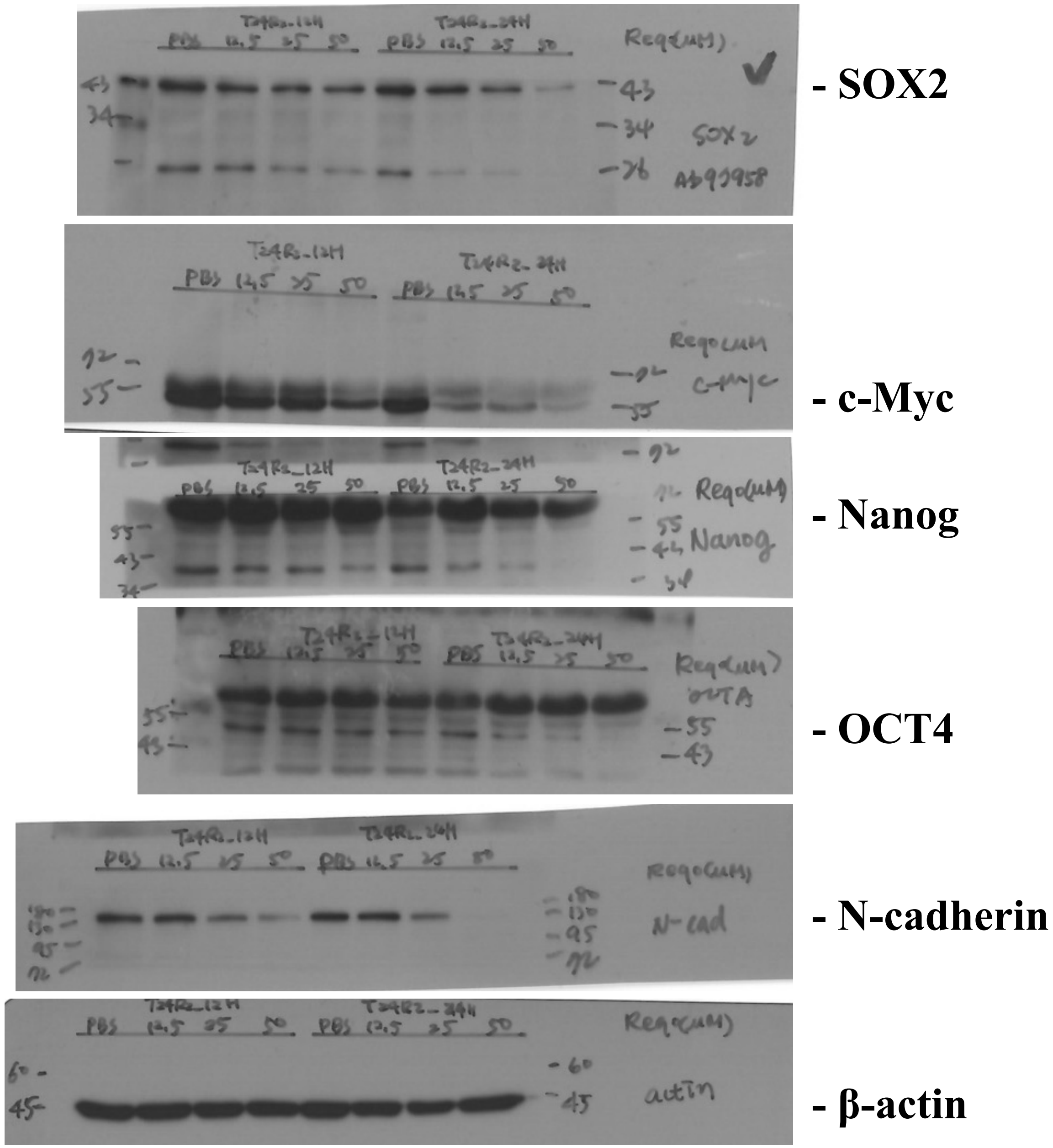

Supplemental Figure 5.
T24R2 raw data of Figure 2E.

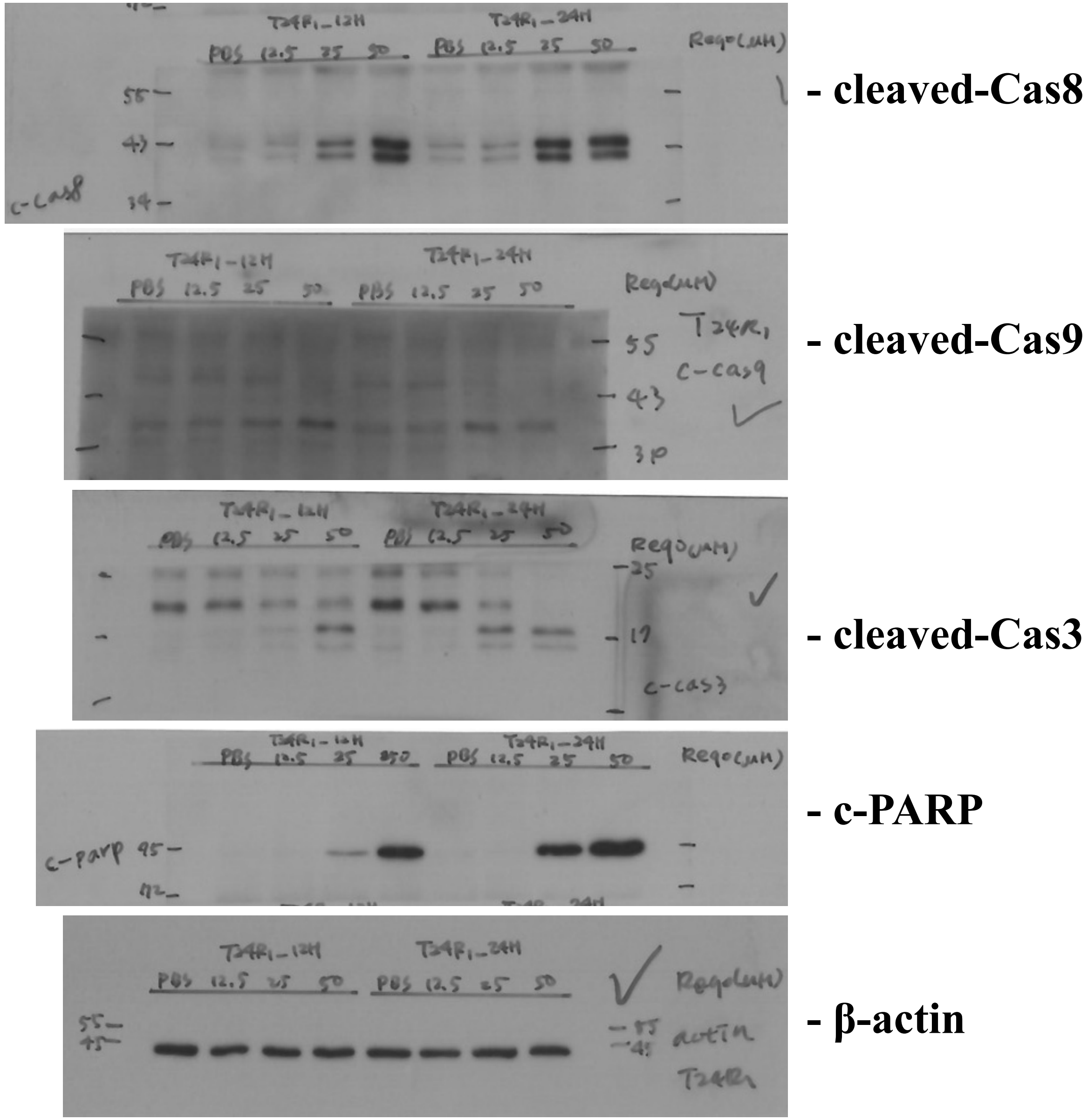

Supplemental Figure 6.
T24R1 raw data of Figure 4B.

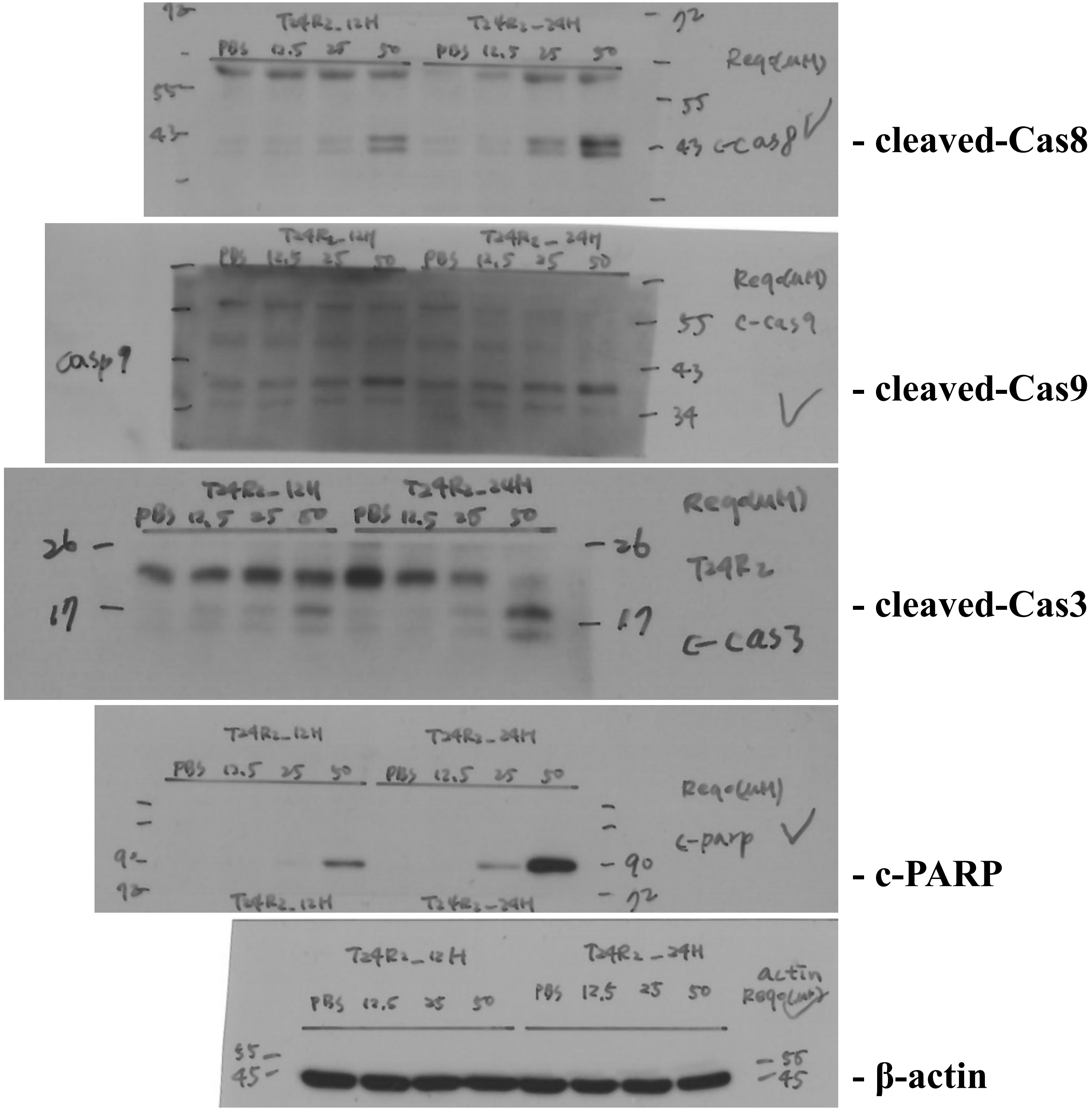

Supplemental Figure 6.
T24R2 raw data of Figure 4B.

T24R1

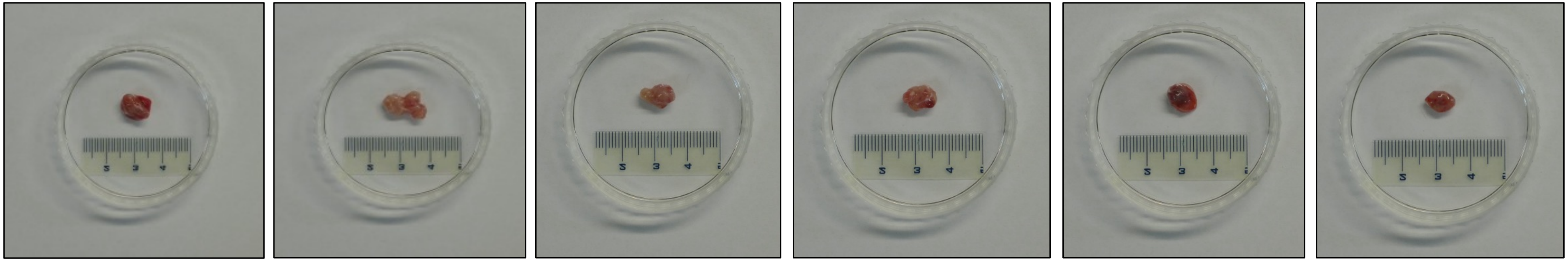

PBS-1 PBS-2 PBS-3 PBS-4 PBS-5 PBS-6

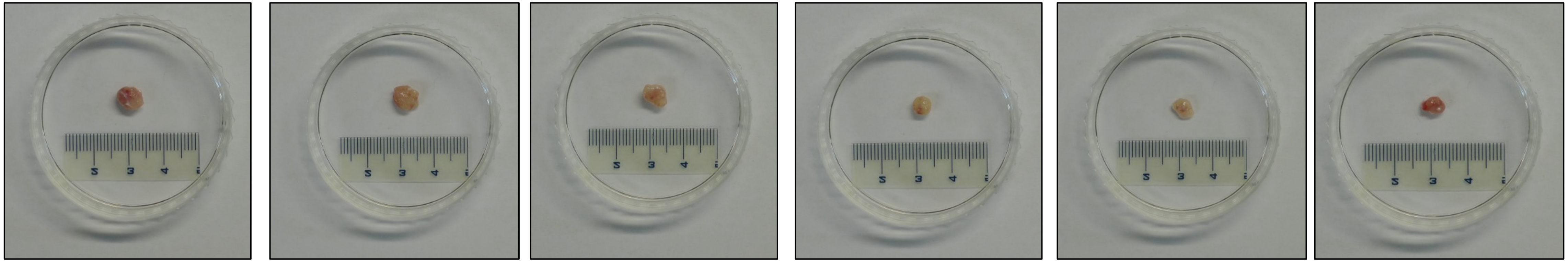

Regorafenib-1 Regorafenib-2 Regorafenib-3 Regorafenib-4 Regorafenib-5 Regorafenib-6

Supplemental Figure 7.
T24R1 raw data of Figure 6A.

T24R2

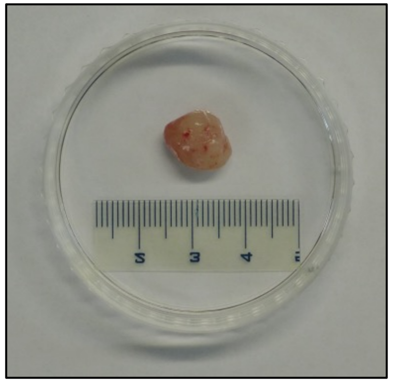
PBS-1

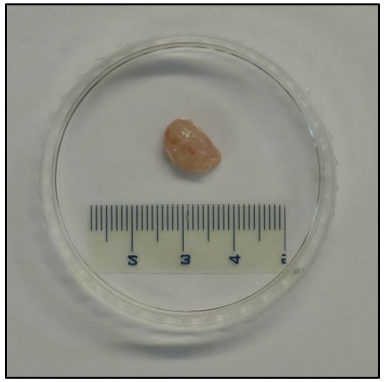
PBS-2

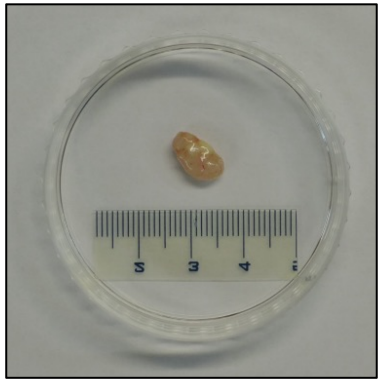
PBS-3

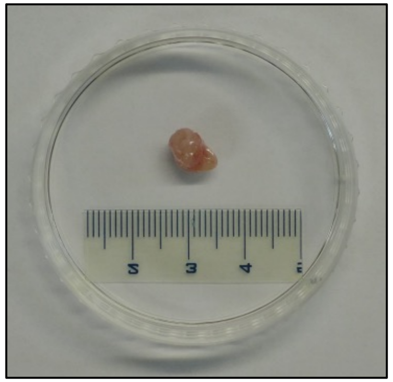
PBS-4

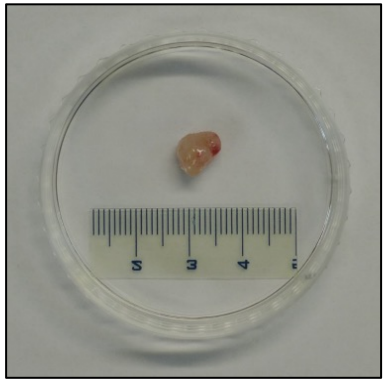
PBS-5

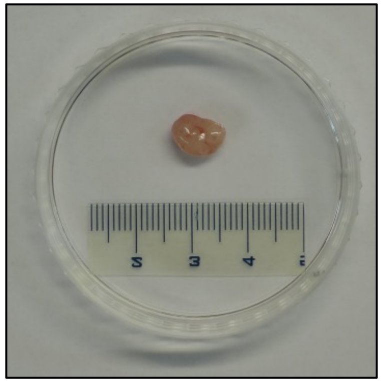
PBS-6

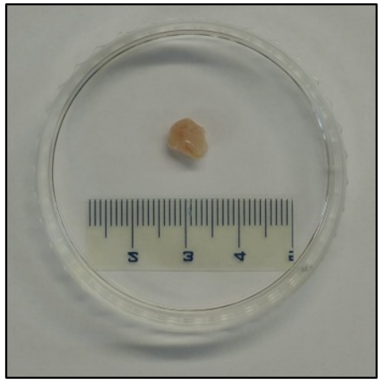
Regorafenib-1

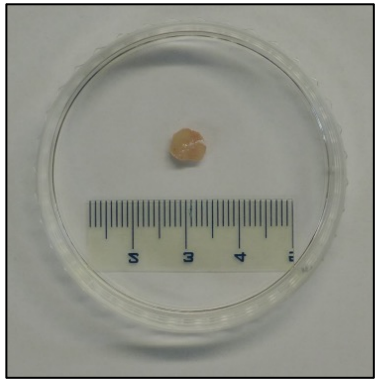
Regorafenib-2

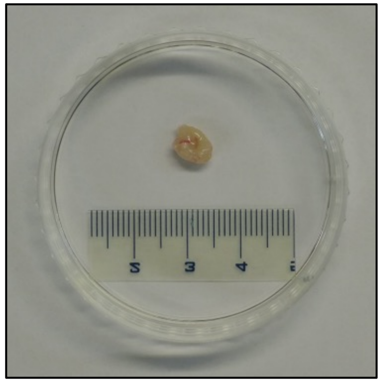
Regorafenib-3

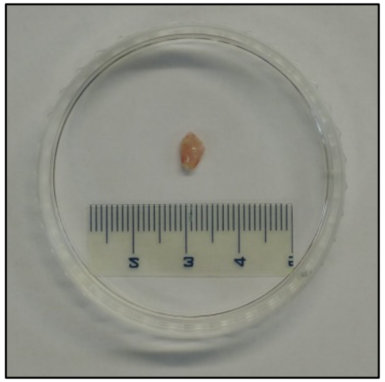
Regorafenib-4

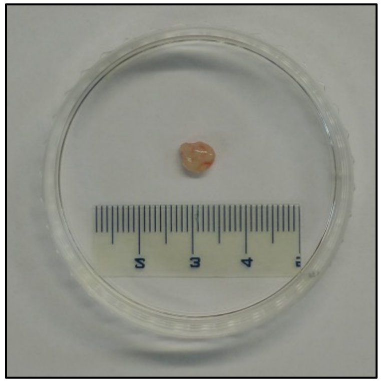
Regorafenib-5

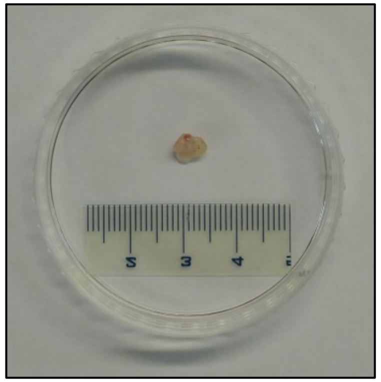
Regorafenib-6

Supplemental Figure 7.
T24R2 raw data of Figure 6A.
